# Fetus-in-Fetu: A Differential Diagnosis of Neonatal Fetiform Encysted Abdominal Mass

**DOI:** 10.7759/cureus.33725

**Published:** 2023-01-12

**Authors:** Muhammad C Jihwaprani, Ahmed A Mousa, Ali A Mohamed, Yousef Alkouz, Inas H Bahlawan

**Affiliations:** 1 Medicine and Surgery, Sulaiman Al Rajhi University, Al Bukayriyah, SAU; 2 Pediatrics, Maternity and Children Hospital, Buraydah, SAU; 3 Obstetrics and Gynecology, Maternity and Children Hospital, Buraydah, SAU; 4 Medicine and Surgery, International University of Africa, Khartoum, SDN

**Keywords:** antenatal diagnosis, twinning, neonatal mass, teratoma, fetus-in-fetu

## Abstract

Fetus-in-fetu (FIF) is a rare embryological anomaly in which an encysted fetiform mass develops within the infant or adult host body. It mainly occurs intraabdominal. There are embryo-pathogenetic debates over whether it belongs to the spectrum of highly differentiated teratomas or is a parasitic twinning from a monozygotic monochorionic diamniotic pregnancy. The presence of vertebral segments and an encapsulating cyst can reliably distinguish FIF from teratoma. The diagnosis may be initially made by imaging modalities such as computed tomography (CT) and magnetic resonance imaging (MRI), and a confirmatory diagnosis made by histopathology of the excised mass. Our center experienced a case of a male neonate presented after emergency cesarean delivery at 40-week gestation with the suspicion of an intraabdominal mass identified antenatally. Antenatal ultrasonography at 34 weeks gestation suggested the presence of an intraabdominal cystic mass measuring 6.5 cm with a hyperechoic focus. A follow-up MRI performed after the delivery showed a well-defined mass with the cystic formation in the left abdominal region with a centrally located fetiform structure. Vertebral bodies and long limb bones were visualized. The diagnosis of FIF was initially made preoperatively by the characteristic findings of imaging studies. Laparotomy was scheduled on day 6, revealing a large encysted mass with fetiform content. FIF should be considered a possible differential diagnosis of neonatal encysted fetiform mass. Routine antenatal imaging permits more frequent antenatal detection with earlier workup and management.

## Introduction

Fetus-in-fetu (FIF) is an extremely rare congenital anomaly in which a vertebrate fetus-like mass is situated within the body of its fully developed host. Two main hypotheses on the pathogenesis of FIF have been described. The "included-twin" or "parasitic-twin" theory suggests that FIF arises from an anomalous monozygotic monochorionic diamniotic twin pregnancy. The malformed fetus becomes entrapped within its twin while dependent on its blood supply via persistent anastomosis of vitelline circulation [1,2]. Alternative theory, the "teratoma-spectrum" theory, suggests that FIF arises as a highly differentiated mature fetiform teratoma. Teratoma is a type of germ cell neoplasm consisting of at least two of three germ layers, i.e. endoderm, mesoderm, and ectoderm. FIF usually presents as a localized swelling that may compress adjacent structures due to the growing mass effect. Diagnosis can be provisionally made by imaging studies such as plain radiography, ultrasonography, computed tomography (CT), and magnetic resonance imaging (MRI), which are confirmed by exploratory laparotomy and complete surgical excision of the mass.

## Case presentation

A full-term male neonate born to a 21-year-old primigravida presented with a swelling in the left abdomen. The baby was previously delivered via emergency cesarean section due to prolonged labor at 40 weeks gestation. Apgar's score was 9 both at 1 and 5 minutes. His birth weight was 3500 grams. The mother did not have any significant previous history of medical illness before and during pregnancy. There was no history of radiation exposure. She was not a smoker. There was no medication history apart from the prescribed antenatal supplements.

During the antenatal period, the mother underwent antenatal ultrasonography (USG) at 29+3-week gestation, which suggested the presence of a congenital anomaly. The USG showed an intraabdominal mass in a single viable developing fetus with a cephalic presentation. The estimated weight of the fetus was 1413 grams and the amniotic fluid level (AFL) was 4.3 centimeters at the largest sack. A follow-up USG was scheduled at 34 weeks of gestation, demonstrating an intra-abdominal cystic mass of unknown origin measuring 6.5 centimeters and a hyperechoic focus measuring 4.5 centimeters. The mass pushed the left kidney anteriorly and medially, resulting in left renal pelvic dilatation. The estimated fetal weight was 2745 grams in cephalic presentation with an AFL of 6.3 centimeters.

**Figure 1:**
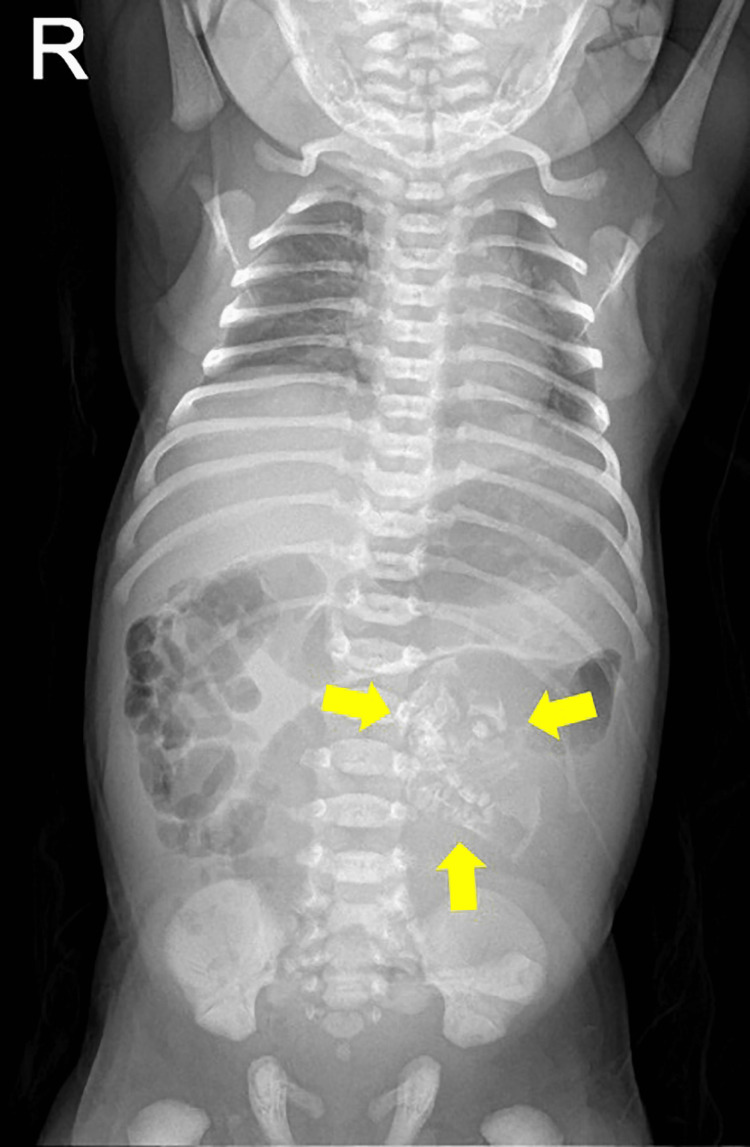
Plain baby-gram X-Ray showing multiple calcifications in the left abdominal area at the level of the L1-L4 vertebrae adjacent to the midline (yellow arrows).

**Figure 2:**
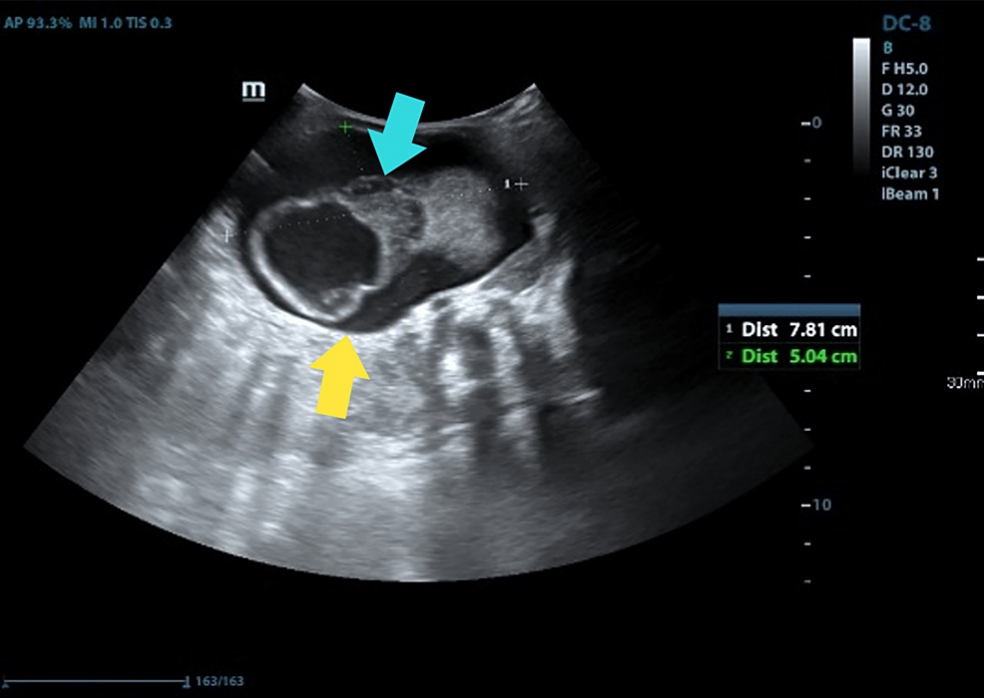
Abdominopelvic USG of the newborn demonstrating a well-defined cystic structure seen in the center of the abdomen to the left side measuring about 80 x 50 millimeters with heterogeneous soft tissues content which is highly suggestive of a nonviable fetus (blue arrow) seen inside the cyst (yellow arrow). Empty skull with vertebral organization and limb buds formation were also visualized. USG: ultrasonography

**Figure 3:**
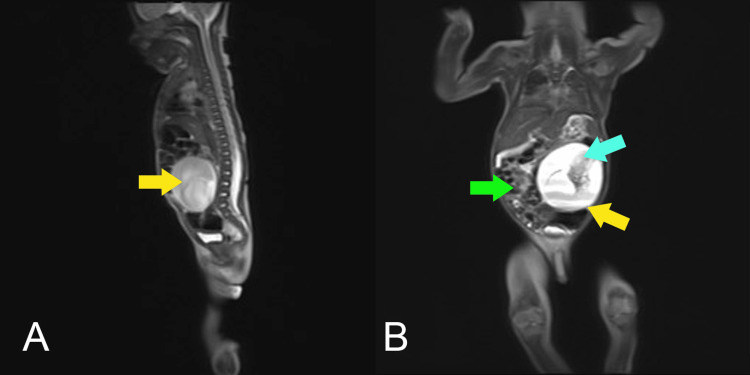
MRI of the patient showing a well-defined cystic mass in the left abdominal region anterior to the left kidney (A and B, yellow arrow) with a centrally located fetiform structure (B, blue arrow). The mass compresses and displaces the bowel loops anteriorly and to the right side (B, green arrow). MRI: magnetic resonance imaging

The complete neonatal examination was remarkable for a distended abdomen with a palpable midline abdominal mass. Laboratory investigation revealed serum α-fetoprotein (AFP) of 3000 nanograms/milliliter (normal: 0-5.8 nanogram/milliliter) and β-human chorionic gonadotropin of 3.16 mIU/liter (normal: 0-5.3 mIU/liter). All other values including complete blood count, urea, electrolytes, and liver function tests were within normal ranges. A plain baby-gram X-Ray (Figure 1) demonstrated a midline abdominal mass with multiple ectopic calcified structures indicating bones. Abdominal USG (Figure 2) revealed a well-defined cystic structure in the center of the abdomen to the left side measuring around 80 x 50 millimeters with heterogenous soft tissue content, which is highly suggestive of a non-viable fetus inside a cyst, with an empty skull, vertebrae, and limbs. Correspondingly, the MRI showed a well-defined cystic mass in the left abdomen anterior to the left kidney with a centrally located fetus (Figure 3 A and B). Multiple long-shaped hyperintensities that correspond to fetal limbs were also observed. Vertebral bodies were visualized on axial T1-weighted MRI which indicates spina bifida. The mass compresses and displaces the bowel loop anteriorly and to the right side (Figure 3 B). The head was not visualized well with a well-defined cystic structure abutting the cervical spine may be due to hydrocephaly or myelomeningocele. Those findings were consistent with a malformed fetus located in the body of its twin, suggesting the diagnosis of FIF.

**Figure 4:**
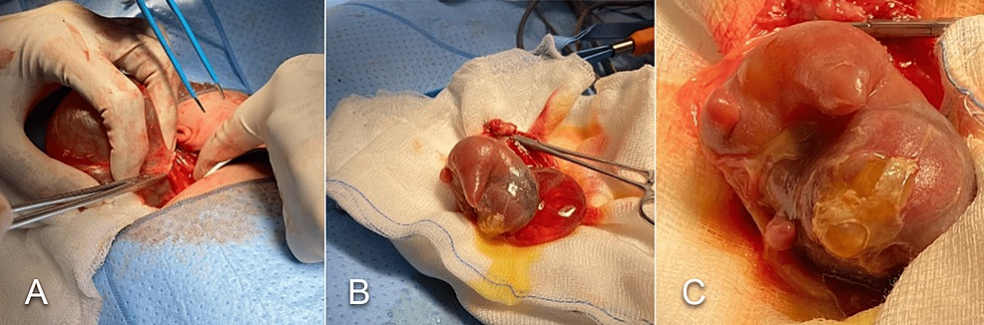
Exploratory laparotomy with excision of the mass revealing a large encysted mass (A). Excision of the cyst showed a fetus-like structure (B and C) with protruding limb buds and anencephalic features.

Exploratory laparotomy was scheduled at the age of six days with a left upper abdominal incision, revealing a large encysted mass (Figure 4 A). The cyst was mobilized from the left colon, left renal vein, and left ureter, and is excised completely. Dissection of the cyst revealed fetus-like structures (Figure 4 B and C) with limb buds visible and anencephalic features. The postoperative period at the neonatal intensive care unit was uneventful, and the baby was discharged at the age of nine days old. A subsequent follow-up visit at six months showed a normally developing infant with no recurrence of the soft tissue mass at the site of surgery. Measurement of serum AFP also demonstrated a regression compared to the preoperative level, measuring around 39 nanograms/milliliter.

## Discussion

FIF is a rare developmental anomaly with a prevalence of less than one in 500,000 births. It has a slight male preponderance (2 male: 1 female) [3]. Despite the two pathogenetic controversies on whether FIF represents a distinct pathogenetic entity (the parasitic-twin theory) or is a part of the fetiform teratoma spectrum (the teratoma-spectrum theory), distinguishing FIF from teratoma is extremely important. That is because even mature cystic teratoma carries a risk of malignancy from 3.5% to as high as 6.67%, while immature teratoma should be regarded as malignant [4,5]. In contrast, there were only two known cases of malignant recurrences in the case of FIF [6,7]. The fetiform mass in FIF is genetically identical to its host body and is usually single, although the case of multiple FIF masses in a single host has also been reported [8]. The differences between FIF and teratoma are summarized in (Table 1).

**Table 1:** Comparison between FIF and teratoma. FIF: fetus-in-fetu

	Fetus-in-fetu	Teratoma
Definition	Congenital anomaly in which a vertebrate fetus-like mass is entrapped within the body of its fully developed host	Germ cell neoplasm due to disorganized pluripotent cell proliferation, contents are derived off of >2/3 germ layers
Common sites	Abdominal, retroperitoneal	Ovaries, testes
Size	Variable, usually 5-8 centimeters^3^	Usually large (>6 centimeters^3^)
Cyst surrounding the fetiform mass	Yes	No
Vascular pedicle	Connected to the host by a small number of relatively large vascular pedicles	No vascular pedicle. Usually has a wider attachment site with multiple small blood vessels.
Anatomical structures	Grossly recognizable, well-formed. Mostly acardiac and anencephalic	Occasional, disorganized. E.g. cartilage, hair, respiratory mucosa, teeth, and intestinal mucosa.
Axial skeletal segments	Its presence is pathognomonic	Not present
Risk of malignant change/recurrence	Extremely low	Immature: High if left untreated Mature: 3.5-6.67%
Staging of the tumor	Not required	Required
α-fetoprotein level	Elevated (typically >3000 nanogram/milliliter)	Not elevated
Management	Surgical excision is curative. An early operation is needed if the mass effect compromises organ functions.	Early surgical excision (risk of malignant transformation if delayed)

Most cases of FIF occur retroperitoneally in the abdominal region (80%). This may be explained by the fact that vitelline circulation embryologically develops into the superior mesenteric artery that is located retroperitoneally. However, its occurrence in other areas has also been reported, including intracranial [9], intrahepatic [10], intrathoracic [11], oral cavity [12], sacrococcygeal [13], and undescended testis [14].

The diagnostic findings favoring FIF over teratoma include the presence of axial skeleton and limb buds on imaging modalities. Willis et al. regarded the presence of a vertebral column as one of the important diagnostic criteria of FIF [15]. The presence of vertebrae indicates an organized embryological development that has developed notochord, the precursor of vertebral bones, during the primitive streak stage. In contrast, teratomata develop as a result of disorganized and uncontrolled pluripotent cell replication, thus vertebral segmentation and organogenesis are not usually found [1,16]. In the neonate, other differential diagnoses should include meconium pseudocyst, which is the result of meconium peritonitis caused by prenatal bowel perforation. In females of reproductive age, ectopic pregnancy should also be considered as a potential differential diagnosis of the fetiform mass [17].

Spencer et al. have proposed that the diagnosis of FIF requires at least one of the following conditions met [2]: (1) it is enclosed within a distinct cyst; (2) it is partially or entirely covered in normal skin; (3) it has anatomical structures that are grossly recognizable; (4) it is connected to the host by a small number of relatively large blood vessels; and (5) it is either positioned immediately next to one of the sites where conjoined twins attached, or be connected to the neural tube or the gastrointestinal system.

Antenatal diagnosis of FIF is made possible by the advancement in imaging modalities. A plain radiograph can sufficiently secure the tentative diagnosis of FIF when the vertebral column and long limb bones are visualized. USG may visualize the fetiform mass and delineate the presence of a cyst enclosing it. More advanced imaging modalities such as CT and MRI can further aid in the diagnosis. In addition to the identification of long bones and vertebral segments, they can establish the exact relationship with the surrounding structures and the mass effect it has caused, e.g. compression and displacement. Laboratory investigations typically reveal an elevated AFP, typically ≥3000 nanograms/milliliter, further supporting the diagnosis of FIF [18]. Consequently, postoperative follow-up for FIF should include the serial measurement of AFP, showing a significant decrease after complete excision of the fetiform mass [19].

## Conclusions

In conclusion, FIF should be considered as a differential diagnosis in a neonate presenting with an encysted fetiform mass. Early diagnosis is made more frequently nowadays due to antenatal routine imaging such as USG. Further imaging modalities such as CT or MRI can reliably differentiate FIF from teratoma by the presence of the encapsulating cyst, vertebral organization, and limb bones in the fetiform mass. The condition should also be differentiated from ectopic pregnancy in adult females and meconium pseudocyst in neonates. Despite its benign nature, post-operative follow-up with serum AFP and imaging modalities may be necessary.
